# PD109 Health Vulnerability: Rethinking Intervention Strategies In A Health Maintenance Organization In Brazil

**DOI:** 10.1017/S0266462324003519

**Published:** 2025-01-07

**Authors:** Matheus del-Valle, Paulo Estevao Franco Braga, Marcus Carvalho Borin, Silvana Marcia Bruschi Kelles, Fernando Martin Biscione

## Abstract

**Introduction:**

This study identified health susceptibility using socioeconomic and environmental indicators, focusing on their impact on population health, by using a health vulnerability index (HVI). The aim was to map vulnerabilities using the HVI among beneficiaries of a health maintenance organization (HMO) in the metropolitan region of Belo Horizonte (MRBH), to enhance access, efficiency, equity, and quality in health services.

**Methods:**

Established in 2012 by Belo Horizonte’s municipality using 2010 census data, the HVI incorporates eight socioeconomic and sanitation indicators. This methodology was extended to all HMO beneficiaries in the MRBH using georeferencing to assess their vulnerability levels (low, medium, high, or very high) and proximity to health facilities.

**Results:**

The findings revealed that 5.44 percent of the HMO’s clients reside in areas categorized as high or very high risk, which corresponds to more than 70,000 individuals. Notably, 91.8 percent of these high-risk beneficiaries are situated in the suburban and peripheral areas of the region and predominantly utilize health facilities located on the outskirts of Belo Horizonte or within the metropolitan area. This distribution underscores a notable disparity in healthcare accessibility and service utilization patterns, with a marked inclination toward the use of emergency services among these populations.

**Conclusions:**

The study underscores a strong correlation between vulnerability and the type of healthcare service utilized, with vulnerable groups often resorting to emergency services, which leads to fragmented care. It highlights the need to improve service processes, particularly for those reliant on public transport, and advocates for an integrated approach to health interventions that promotes equitable healthcare access in complex socioeconomic landscapes.

